# Is beauty beyond the eye of the butterfly?

**DOI:** 10.1371/journal.pbio.3003054

**Published:** 2025-03-12

**Authors:** Richard M. Merrill

**Affiliations:** Faculty of Biology, LMU, Munich, Germany

## Abstract

The diversity of bright colors observed across the animal world are often used during mate choice. This Primer explores a new study in PLOS Biology which reveals genetic and neural mechanisms contributing to the evolution of visual mating decisions in Heliconius butterflies

## Teaser

The bright colors observed across the animal world are often used during mate choice. An exciting new study in *PLOS Biology* suggests genetic and neural mechanisms contributing to the evolution of visual mating decisions in *Heliconius* butterflies.

Animals often use color to inform their decisions. Although color discrimination is increasingly well-characterized at the molecular level [e.g. 1], we still know little about the genetic and neural changes underlying the evolution of how animals respond to visual cues. This is important because how selection shapes behavioral variation may depend on the mechanisms involved, including the relationship between reception and response. For example, while genetic changes influencing receptor sensitivity may avoid potentially deleterious modifications to downstream circuits, they might also alter perception of the broader environment on which multiple behaviors rely. This has been hypothesized to drive differences in mate choice [2], but in other situations it might equally act as a constraint. In an impressive marriage of evolutionary genomics and neurobiology, a recent *PLoS Biology* study by VanKuren and colleagues [3] begins to unravel the genetic and neural mechanisms underlying visual mate choice evolution in a tropical butterfly.

The bright wing patterns of *Heliconius* butterflies primarily function to warn birds that they are distasteful, but male *Heliconius* also use them to identify suitable mates. Closely related *Heliconius* species often display divergent wing patterns, and because males almost invariably prefer to court females that share their own colors, this can help maintain the integrity of taxonomic boundaries. Individuals of the same species tend to share the same pattern (at least within a single geographic location); however, some populations, including one of the *H. cydno* populations studied by VanKuren and colleagues, are polymorphic. Specifically, in western Ecuador, individual *H. cydno alithea* has either a white or yellow forewing patch. Previous work has revealed that these differences in warning coloration are coupled with corresponding mating preferences, so that yellow males preferentially court yellow females (white males appear to have no preference) [4].

Coupling of ecological and mating traits is often considered an efficient route towards speciation. This is because it allows divergent selection acting on the ecological trait to maintain variation in the mating trait, but determining the exact genetic mechanisms involved has often proved difficult (reviewed in [5]). Possible scenarios include pleiotropy, where the same allele influences variation in two seemingly unrelated phenotypes, as well as tight linkage, involving physically close but distinct loci on the same chromosome. Associations may also arise through population-level processes [6]. For example, because males with a preference for yellow are more likely to pair with yellow females, they will consequently also be more likely to sire offspring carrying both the yellow preference allele *and* the yellow color allele (even if the loci are on different chromosomes).

**Fig 1:**
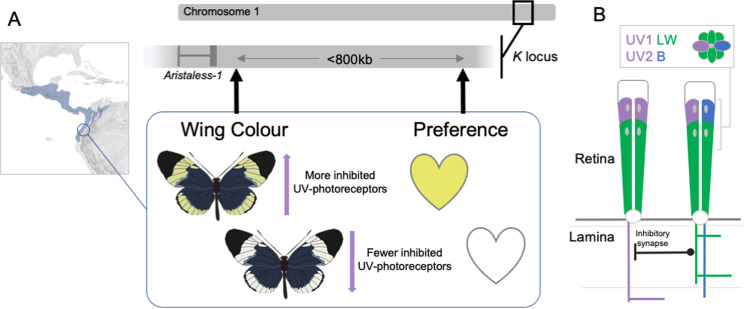
Wing color is coupled with the corresponding male mating preference due to the action of physically close but distinct loci, and also correlates with UV-photoreceptor inhibition. **A)** Using genome-wide association analyses, VanKuren and colleagues [3] show that separate loci within the K locus control a white-yellow color switch and contribute to variation in male preference, respectively, in a polymorphic population of *Heliconius cydno* in Ecuador. The full geographical range of *H. cydno* is shown on the map in light blue. The approximate location of the color pattern gene *Aristaless-1* [7] is shown for reference; other genes (not shown) are also present in the K locus, including some that are differentially expressed during the development of visual processing regions of the insect brain. In this and other *H. cydno* populations, white-yellow wing coloration correlates with the proportion of inhibited UV-photoreceptors, suggesting a compelling mechanism for the evolution of visual preference. Base maps were obtained from Herwig Schutzler https://www.shadedreliefarchive.com. **B)** Individual ommatidia in the butterfly eye are composed of multiple photoreceptors. Inter-photoreceptor synaptic connections are known to exist within the lamina, the upper layer of the optic lobe, itself the region of the insect brain responsible for initial visual processing.

To address this, VanKuren and colleagues sequenced 113 *H. cydno alithea* males sampled from the polymorphic population in Ecuador, previously assayed for their relative preference for yellow or white females [4]. Genome-wide association (GWA) analyses subsequently revealed four regions in the genome associated with mating preference. Importantly the best supported locus was < 800 kb away—though still distinct—from the top hit for the white-yellow forewing color switch (within a region on chromosome 1 referred to as the ‘K locus’ and containing the color pattern gene *aristaless-1* [7]) (Fig 1). This example of tight linkage broadly mirrors the situation observed between *Heliconius* species separated by a white-to-red forewing switch, where physically close but distinct genes, *optix* and *regucalcin1*, respectively, regulate wing color and the corresponding behavioral preference for either white or red females [8].

To ask how genetic variation might be translated into the white-yellow preference differences observed in *H. cydno*, VanKuren and colleagues recorded the response of individual photoreceptors to different wavelengths of light. This allowed them to specifically test whether variation in wing coloration (as a proxy for behavioral preference) correlates with differences in photoreceptor tuning, implying a direct link between reception and response. Although they found that photoreceptor sensitivity does indeed vary between *Heliconius* populations, and argue that this might aid visual discrimination in some contexts, the correlation between UV-photoreceptor spectral sensitivity and wing color did not hold in the polymorphic population from Ecuador. This suggests that variation in photoreceptor tuning is unlikely to be the primary cause of differences in mating preferences in this case. However, subsequent experiments revealed that some UV-sensitive photoreceptors in male *Heliconius* eyes receive inhibitory input from long-wavelength-sensitive photoreceptors, as has been observed in other insects. Remarkably, the proportion of inhibited UV photoreceptors is correlated with wing color, including within the key polymorphic population in Ecuador.

Previous studies in *Papilio* butterflies have shown that inhibitory synapses occur in the axons within the lamina, the upper layer of the optic lobe, the region of the insect brain responsible for initial visual processing [9]. Although VanKuren and colleagues have not yet explicitly linked the expression of specific genes to behavioral variation, they identify promising candidates, notably including some differentially expressed in the developing optic lobe. This will undoubtedly guide future work to visualize the expression of these genes in relation to the development of inhibitory synapses, and to experimentally test their effect on behavior. Regardless, the striking correlation between variation at the *K* locus and UV-photoreceptor inhibition, both within and between populations of *H. cydno*, already provides a new and compelling mechanistic hypothesis for the evolution of visual preferences.

Overall, results of this study suggest a model where variation in yellow-white *Heliconius* mating preferences depends on changes in downstream signal processing, rather than sensory reception. Of course, questions remain. For example, how are preferences for other types of color and pattern variation encoded in the sensory systems of *Heliconius*, and elsewhere? How often is the coupling of key traits of maintained through tight linkage, as opposed to other mechanisms such as pleiotropy, which is perhaps a more convincing hypothesis in other systems [e.g. 10]. And might the evolution of visual mate choice depend less often on changes in sensory reception than other modalities, such as olfaction (maybe due to the larger number and increased specificity of chemoreceptors on which selection can act)? Nevertheless, despite these and many other potential avenues for future research, this work by VanKuren and colleagues makes an important contribution to our expanding vision of behavioral evolution.
